# Dental Implant Treatment With Guided Bone Regeneration in a Patient With Active Crohn’s Disease Under Long-Term Immunosuppressive and Anti-inflammatory Therapy

**DOI:** 10.7759/cureus.83439

**Published:** 2025-05-04

**Authors:** Krasimir Chapanov, Daniela Stoeva, Aleksandar Naydenov

**Affiliations:** 1 Department of Dental Implantology, Medical University-Sofia, Sofia, BGR; 2 Department of Prosthetic Dental Medicine, Medical University-Sofia, Sofia, BGR

**Keywords:** anti-inflammatory, autoimmune disease, bone regeneration, immunosuppression, implantology

## Abstract

This report presents a case of dental implant treatment with guided bone regeneration in a 46-year-old patient with active Crohn’s disease, who was undergoing long-term immunosuppressive therapy (azathioprine) and anti-inflammatory treatment (mesalazine). A titanium implant was placed six weeks after the extraction of tooth 22. Alveolar augmentation was performed using synthetic xenograft material in combination with a collagen membrane. Seven months later, a provisional restoration was delivered, followed by the placement of a definitive screw-retained crown 120 days afterward. No complications were reported throughout the treatment, and the patient consistently maintained excellent oral hygiene. This case underscores the feasibility of implant therapy in immunocompromised patients, despite longstanding concerns regarding the potential negative impact of immunosuppressive agents on bone metabolism and healing. Treatment success relied on a thorough medical history review, precise planning, minimally invasive surgical techniques, and the use of specific postoperative medications. Regular follow-up visits and diligent oral hygiene were also key to the favorable outcome. While this individual case was successful, further clinical studies are needed to establish comprehensive guidelines for implant treatment in patients with autoimmune conditions or organ transplants who are receiving immunosuppressive and systemic anti-inflammatory therapy.

## Introduction

Dental implants demonstrate a high long-term success rate in replacing missing teeth, with cumulative survival rates of 92% for two-stage implants over 15 years and 85% for one-stage surgery implants over 10 years, including early failures [1]. However, certain patients present a higher risk of treatment failure due to serious systemic diseases or ongoing medication use. In such cases, both early and late complications may arise during routine dental procedures as well as surgical interventions like dental implant placement.

To sustain long-term success in implant therapy, a comprehensive assessment is essential. This includes a detailed review of the patient’s medical history, the presence of controlled or uncontrolled systemic diseases, long-term medication use, and drugs with prolonged half-lives. Understanding these factors is crucial in planning a safe and effective treatment.

The goal of dental implant therapy is to achieve successful and long-lasting outcomes with minimal complications. Ideally, the treatment process should be as short and non-invasive as possible. Nonetheless, systemic diseases and related medications can negatively impact treatment results. Drugs such as anti-inflammatories, immunosuppressants, and immune modulators can interfere with bone metabolism and healing, potentially compromising the outcome of procedures like dental implant placement and bone augmentation [2].

Despite these risks, advances in dental implantology have made it possible to achieve successful results even in complex clinical scenarios. However, in 2016, Radzewski and Osmola emphasized that implant placement in certain patient groups remains controversial. This includes individuals undergoing immunosuppressive therapy for conditions such as rheumatic diseases, autoimmune skin disorders, or organ transplants [3].

There is limited clinical evidence supporting these concerns. In fact, several studies investigating the effects of immunosuppressive therapy on dental implants found no negative impact on treatment outcomes [4-6]. For example, patients awaiting organ transplantation often undergo dental extractions to eliminate potential sources of infection. Given the improved survival rates and quality of life for transplant recipients, restoring proper masticatory function becomes a priority. Thus, replacing missing teeth with dental implants plays a significant role in post-transplant rehabilitation.

Approximately two decades ago, immunosuppression was considered an absolute contraindication to implant surgery due to concerns about drug-induced bone toxicity [7]. However, this position was not supported by direct evidence. While some studies confirm that immunosuppressive drugs can negatively affect bone healing [8], the process of osseointegration itself remains stable, even when immunosuppressive therapy is modified or discontinued.

Immunosuppressants are commonly used to suppress or eliminate immune responses, primarily to prevent rejection of transplanted organs and to manage autoimmune diseases. These drugs, although effective, are associated with various side effects. They include corticosteroids such as prednisone, budesonide, and prednisolone; calcineurin inhibitors like cyclosporine, tacrolimus, and pimecrolimus; mTOR inhibitors including sirolimus and everolimus; IMDH inhibitors such as azathioprine, leflunomide, and mycophenolate; as well as biological drugs and monoclonal antibodies like adalimumab, etanercept, trastuzumab, interferon beta-1a, ranibizumab, ixekizumab, secukinumab, basiliximab, daclizumab, and muromonab [9].

Cyclosporine (CsA) is a well-known immunosuppressive agent. Several studies have examined its influence on osseointegration, yielding different conclusions over time. In 2001, Duarte et al. reported that the combination of CsA and nifedipine negatively affected bone healing in tibial implants in rabbits [8]. However, in a 2003 follow-up study using a similar design, the same researchers found that short-term therapy with these drugs might not significantly impact osseointegration [10].

Further evidence supporting the feasibility of implant therapy in immunosuppressed patients comes from a 2011 study by Gu and Yu. The researchers investigated 13 patients who had undergone liver transplantation and were on long-term immunosuppression. A total of 45 dental implants were placed, and the patients received drugs such as tacrolimus, mycophenolate mofetil, cyclosporine, and prednisone. Implant loading occurred after three months in the mandible and six months in the maxilla. During the three-year follow-up, no implants were lost [11]. This study suggests that, with consistent professional monitoring and good oral hygiene, patients on immunosuppressive therapy or those who have received organ transplants can successfully receive dental implants.

## Case presentation

A 46-year-old woman visited our clinic for consultation and treatment. During the clinical examination, it was determined that tooth 22 had a poor prognosis due to a longitudinal fracture of both the crown and root. Conservative and conservative surgical treatments were not optimal. The tooth exhibited 2 mm of mobility in the buccolingual direction (Miller classification). The patient maintained excellent oral hygiene, with no inflammation observed in the oral mucosa. She was a non-smoker and did not consume alcohol. The adjacent teeth and antagonists were vital. The patient demonstrated stable socioeconomic status, ensuring consistent access to dental care and adherence to postoperative follow-up protocols.

In 2020, the patient was diagnosed with active Crohn’s disease following a colonoscopy, during which biopsies from the terminal ileum and colon revealed transmural inflammation and non-caseating granulomas, confirming the diagnosis histologically. She had reported lower-dyspeptic symptoms, including abdominal pain and diarrhea, since 2019, which were consistent with active disease. The patient provided comprehensive medical records, including pathology and endoscopy reports, confirming the diagnosis of active Crohn’s disease. She reported taking IMURAN™ 50 mg tablets (azathioprine, an immunosuppressant) and Salofalk 500 mg (mesalazine, an anti-inflammatory drug used to treat inflammatory bowel disease). The patient had no other systemic comorbidities.

**Figure 1:**
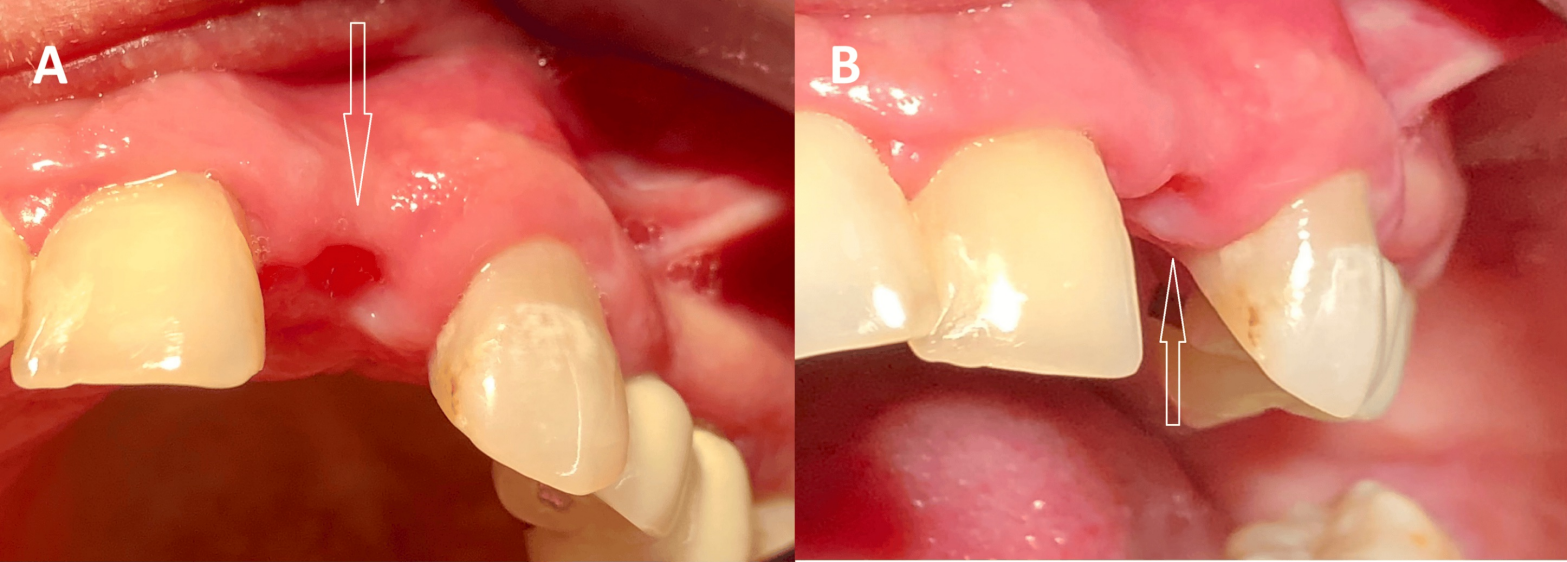
Two months after the extraction of tooth 22 (A, B) The soft tissues in the extraction site show complete healing.

Given that the clinical symptoms had started six months prior and considering both local and general health factors, the following treatment plan was proposed: extraction of tooth 22 and early implant placement (four to eight weeks after extraction), followed by a conventional loading protocol (with a healing period of more than two months after implant placement). An uncomplicated, low-trauma extraction (vertical tooth extraction without the use of luxation) of tooth 22 was performed. After two months, soft tissue healing was assessed, and dental implant placement was planned (Figure 1).

**Figure 2:**
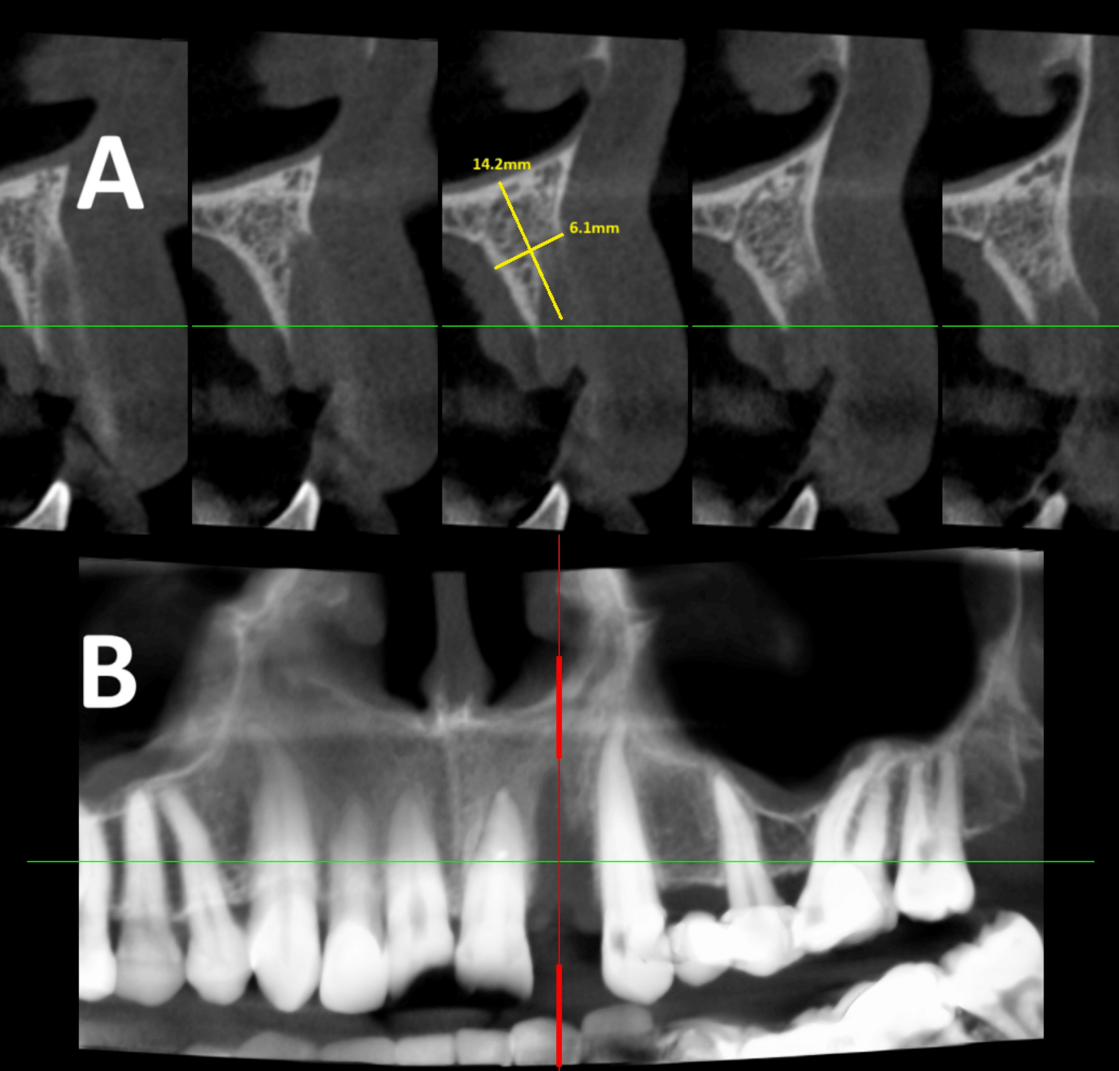
(A) Sagittal and (B) coronal views of CBCT images before implant placement Bone volume measurements are highlighted in yellow. CBCT, cone beam CT

**Figure 3:**
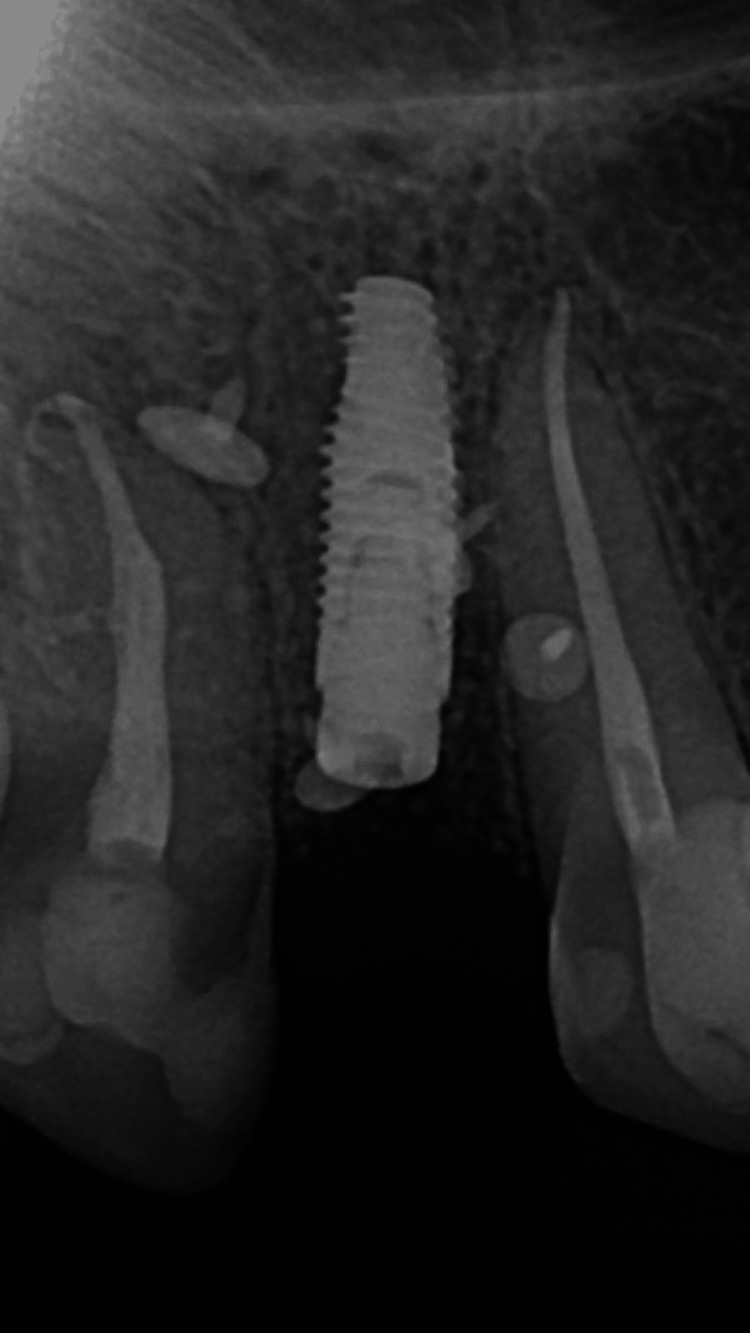
Periapical X-ray following implant placement and guided bone regeneration Titanium pins used to secure the collagen membrane are visible around the implant.

Additional X-rays were taken, revealing both horizontal and vertical bone defects, vestibular to the area of the missing tooth. The bone volume was insufficient, preventing proper 3D positioning of the implant (Figure 2). As a result, the planned early implant placement was modified, and guided bone regeneration was performed in the area (Figure 3).

After preparation of a mucoperiosteal flap, a dental implant with a diameter of 3.5 mm and a length of 10 mm was placed with a torque above 45 Ncm to achieve high primary stability (Helix GM, Neodent, Curitiba, Brazil). A synthetic xenograft (Maxresorb, botiss biomaterials GmbH, Zossen, Germany) was used to fill the vestibular bone defect. The graft was covered with a pericardial collagen membrane (15 × 20 mm, Jason, botiss biomaterials GmbH) and secured with titanium pins (botiss biomaterials GmbH). The soft tissues were carefully adapted and sutured without tension. Postoperative medication included analgesics (400 mg MIG-400, twice daily for seven days), mouthwash (0.1% Chlorhexidine, Eludril Classic, Pierre Fabre S.A., Paris, France; three times daily for 14 days), and an antibiotic (Ospamox 1000 mg, Sandoz GmbH, Kundl, Austria; twice daily for seven days). There were no complications or complaints during the postoperative period.

**Figure 4:**
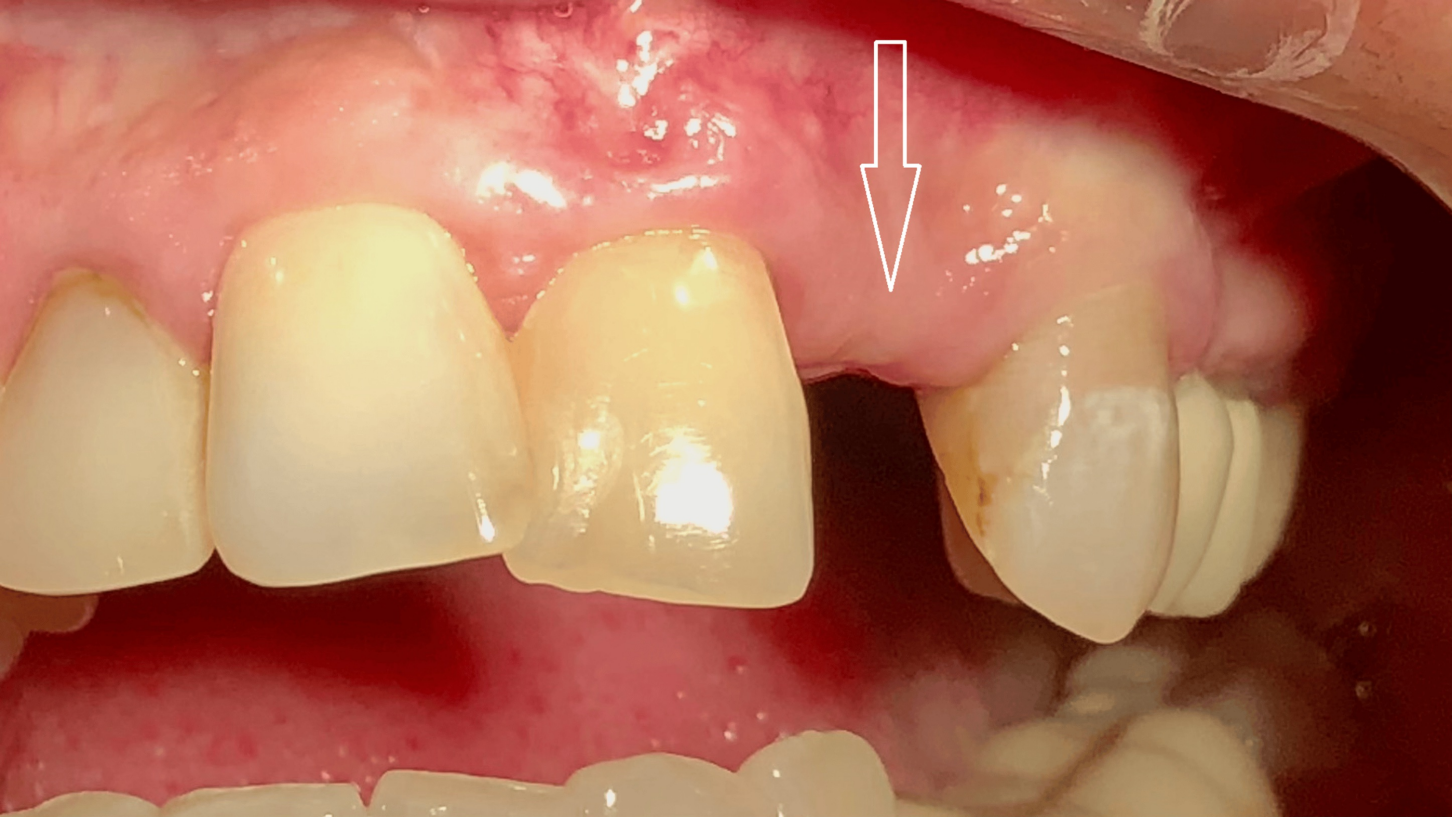
Intraoral view seven months after implant placement and guided bone regeneration The soft tissues appear healthy, with no signs of inflammation.

**Figure 5:**
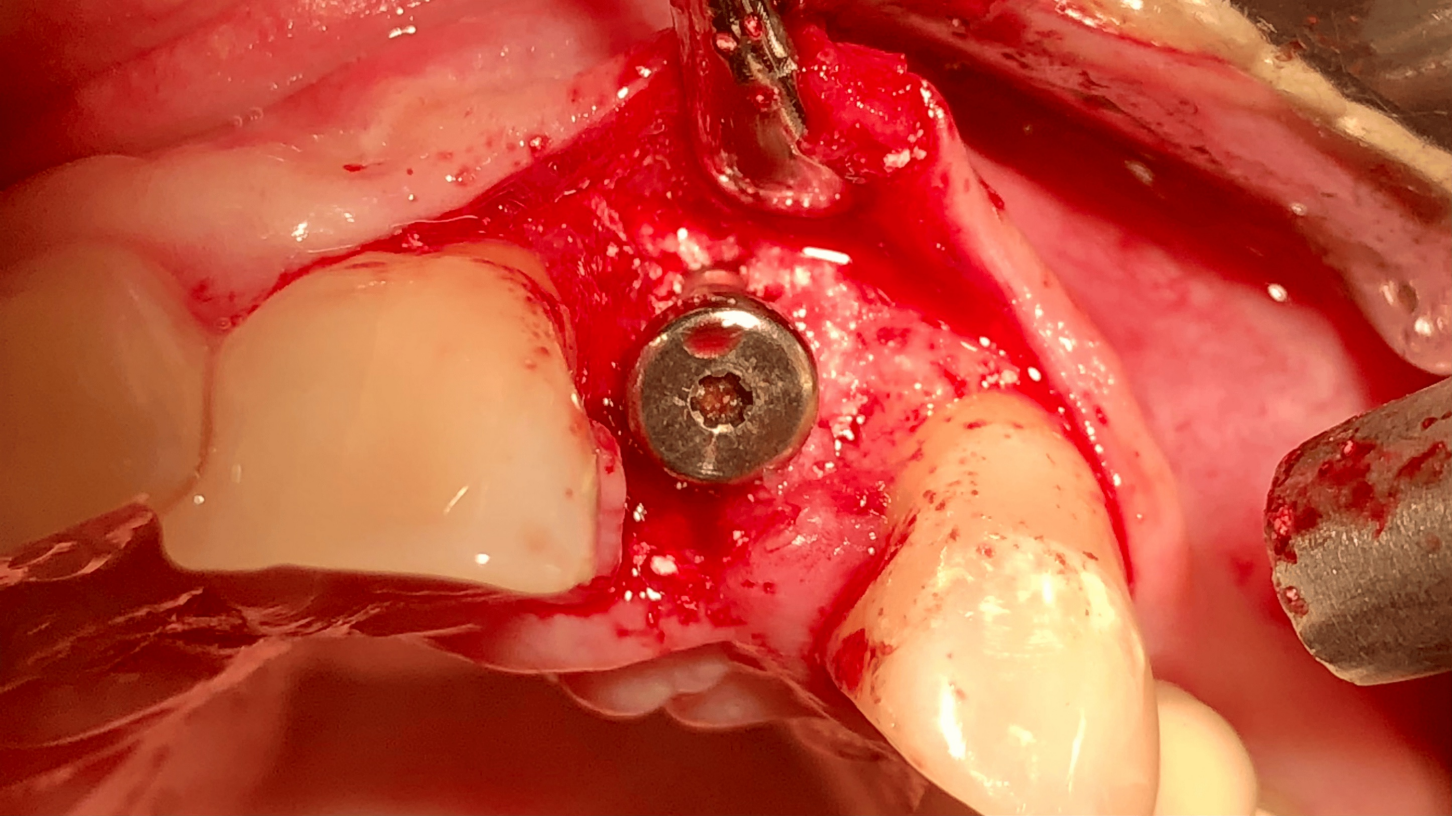
Intraoral view during the surgical exposure of the implant The bone substitute particles in the crestal zone are well integrated.

**Figure 6:**
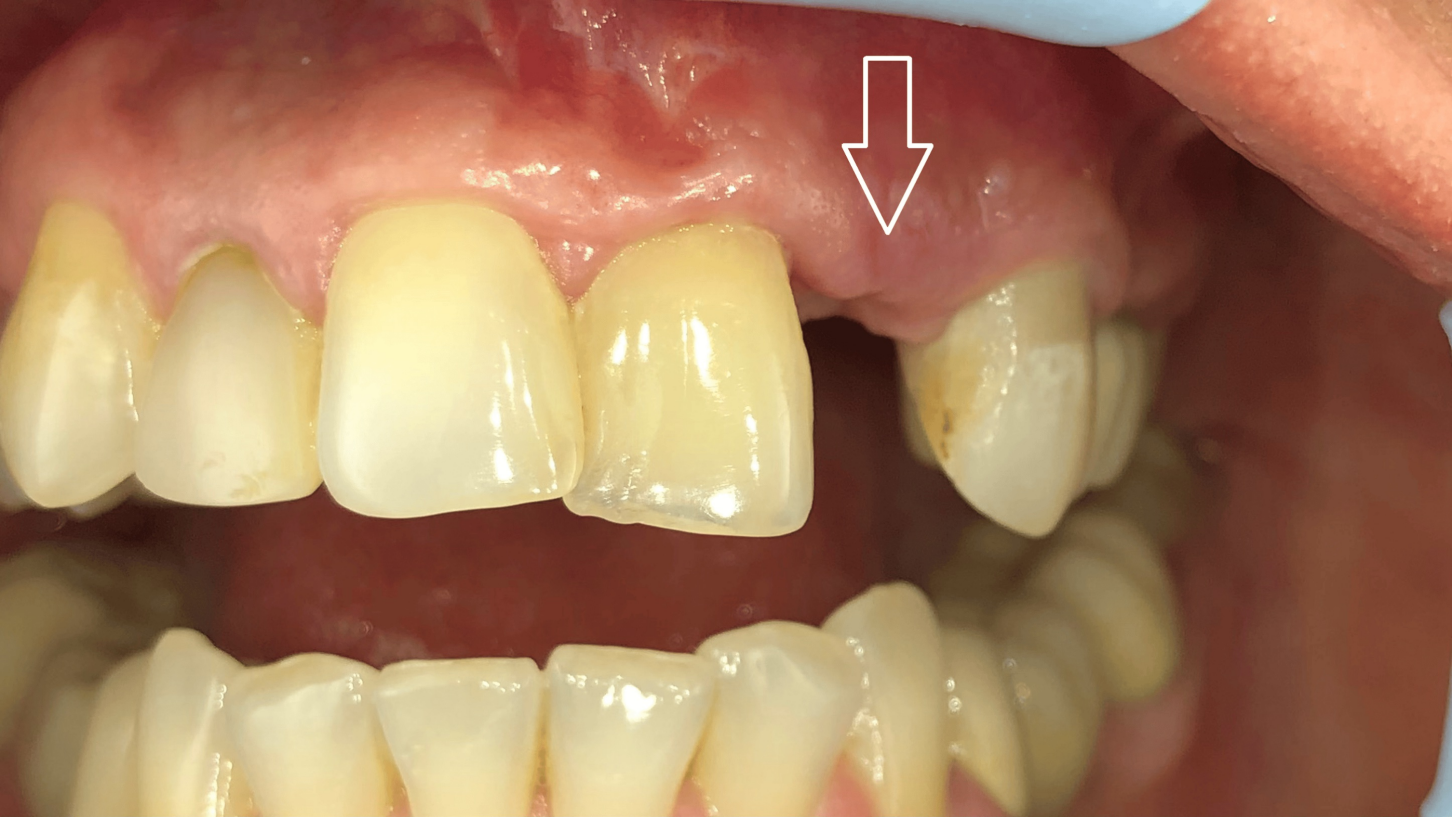
Intraoral view 20 days after implant uncovering The healing screw is positioned below the level of the well-healed soft tissues.

**Figure 7:**
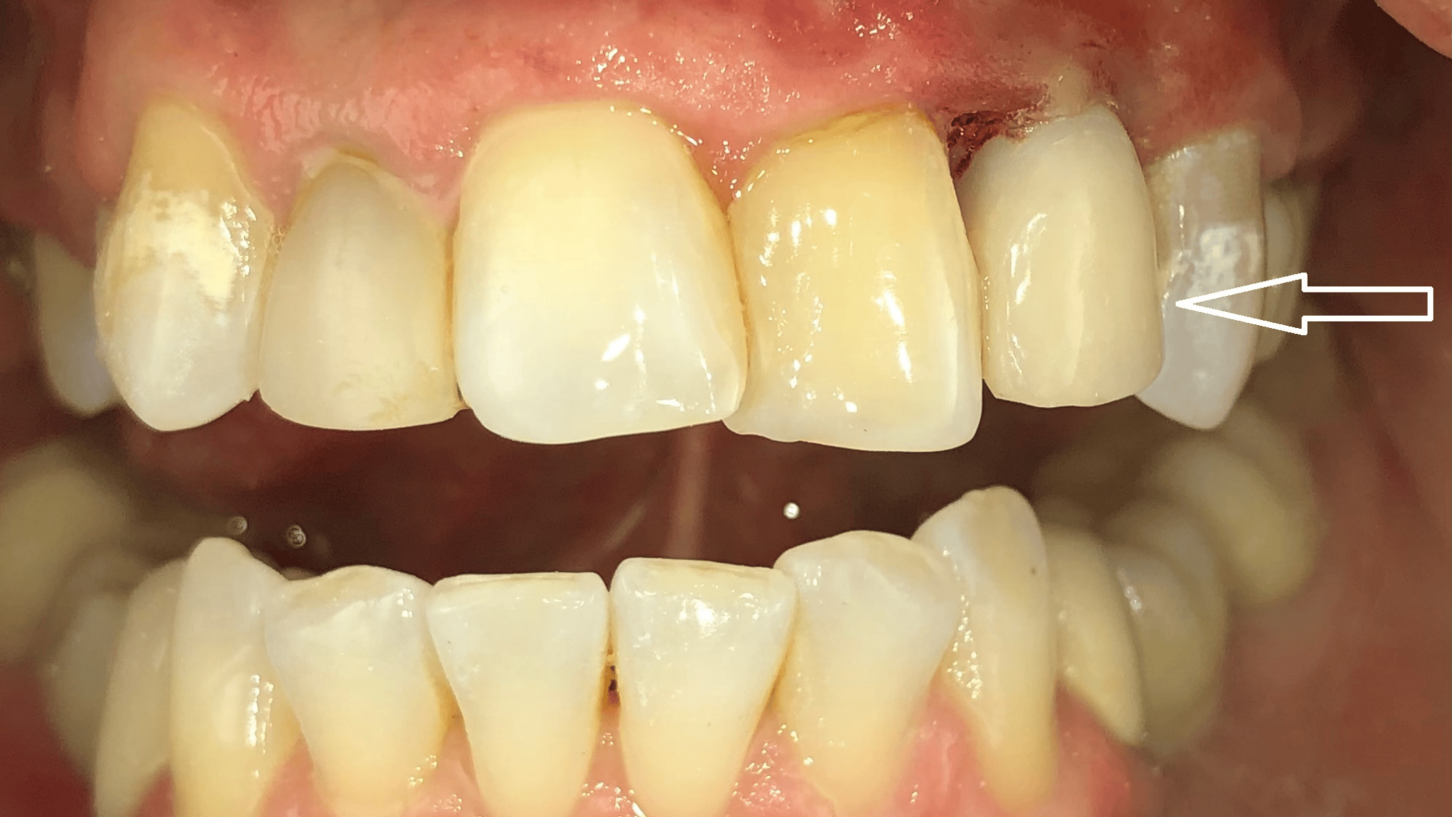
Intraoral view on the day of fixation of the screw-retained provisional crown

Seven months later, an assessment and evaluation of the soft tissues and bone augmentation around the implant were conducted. An individual provisional restoration was then fabricated (Figure 4, Figure 5, Figure 6, Figure 7).

**Figure 8:**
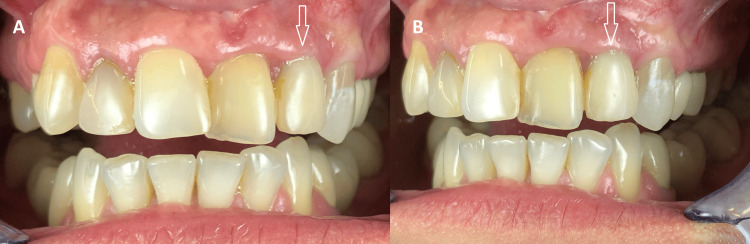
(A, B) Intraoral view 120 days after fixation of the provisional crown Well-defined soft tissue healing over the crown is observed.

**Figure 9:**
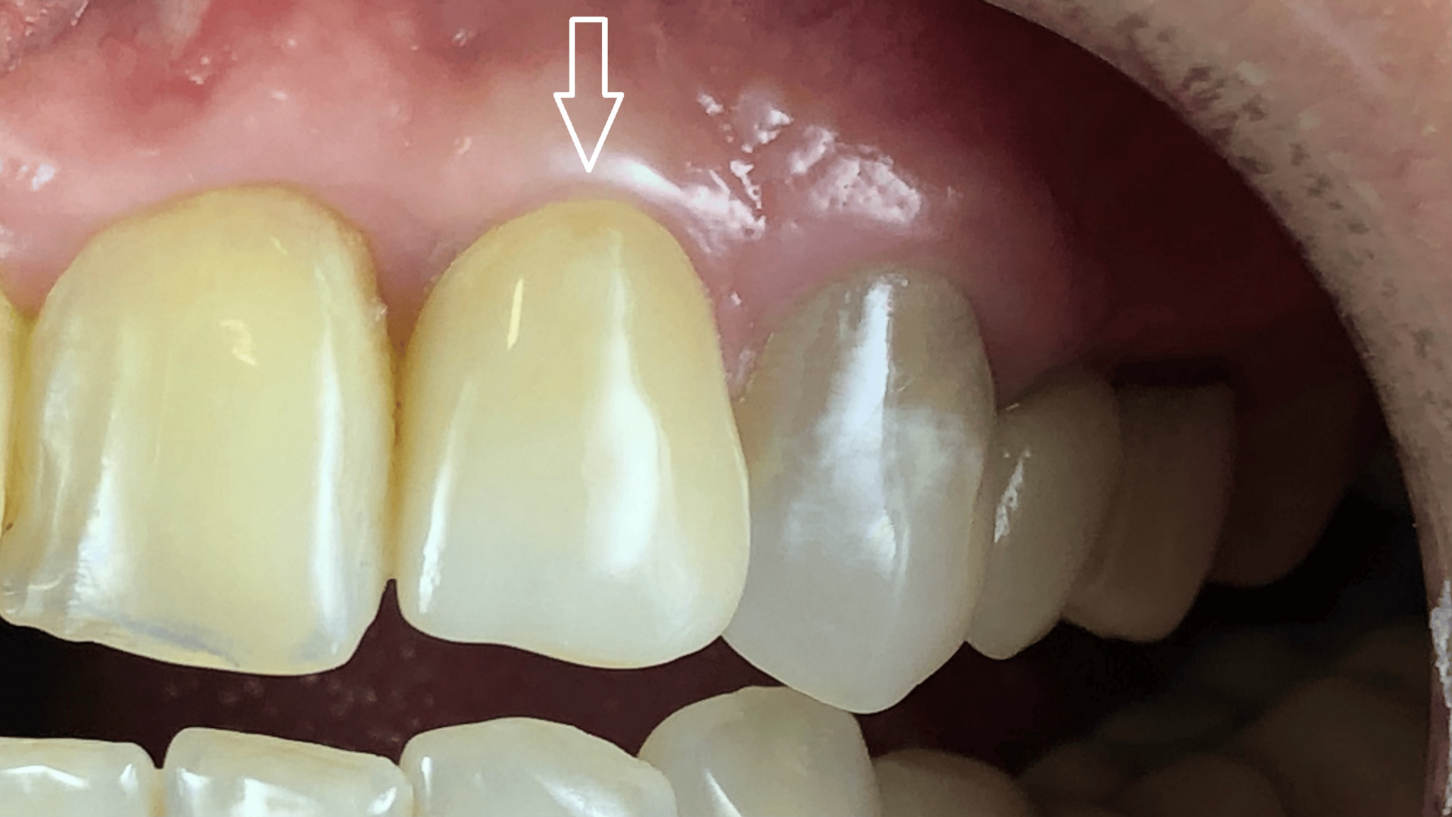
Intraoral view one month after fixation of the permanent zirconia crown Stable soft tissue volume and contour are observed around the implant, with no clinical evidence of peri-implant mucositis or peri-implantitis.

Over a period of 120 days, the patient did not report any issues with maintaining oral hygiene around the provisional crown, nor was there any impact from food debris. As a result, no adjustments to the provisional crown were necessary. We proceeded with the conventional impression technique and fabricated a zirconia crown, which was cemented onto a titanium base (Ti-base, Neodent) (Figure 8, Figure 9). The patient was provided with instructions for maintaining good oral hygiene around both the implant and the remaining dentition. A regular follow-up plan was also discussed.

## Discussion

Patients with autoimmune diseases or those who have undergone organ transplants rely on immunosuppressive drugs for life to prevent disease progression or organ rejection. The use of dental implants and/or guided bone regeneration of the alveolar process presents a significant challenge in these patients, requiring careful consideration of each case to optimize treatment outcomes. Most immunosuppressive drugs affect the entire immune system, which increases the risk of side effects. By altering the immune system, these drugs make patients more vulnerable to postoperative infectious complications [12]. For patients on long-term corticosteroid therapy, it is advisable to assess the possibility of adrenal insufficiency [13]. Glucocorticoids, which have potent anti-inflammatory and immunosuppressive effects, are commonly used to treat inflammatory and autoimmune diseases. These drugs modify anabolic processes and suppress immune function, potentially compromising implant treatment. Chronic glucocorticoid use can also lead to steroid-induced osteoporosis and elevated serum glucose levels [14].

Cytostatics, often prescribed for patients with malignancies, do not differentiate between malignant and normal cells, making them cytotoxic to healthy tissues. Most chemotherapeutic agents have cytotoxic effects on bones, particularly in grafts with insufficient or disrupted blood supply. Chemotherapeutic drugs have a strong affinity for cells with high turnover rates [15], which often affects the oral mucosa, leading to clinical manifestations such as mucosal ulcers.

Cyclosporine, another immunosuppressive drug, can negatively impact bone around implants, potentially compromising primary stability and osseointegration [16]. Radzewski and Osmola emphasized the importance of adjusting medication regimens, noting that the use of newer immunosuppressive drugs with lower toxicity is a significant factor in improving outcomes [3].

With ongoing pharmacological advancements, adjustments to drug therapy should be considered during the treatment of autoimmune diseases or in transplant patients. Over the years, calcineurin inhibitors like tacrolimus have been used with reduced toxicity to bones.

Effective implant planning requires a comprehensive understanding of the patient's medical history, including past and present conditions. Combining appropriate medication with minimally invasive surgery can minimize the risk of postoperative complications. Additionally, regular checkups, professional oral hygiene, and patient motivation are essential for ensuring treatment success.

## Conclusions

Although immunosuppressive drugs hinder bone healing, patients with autoimmune diseases can successfully undergo dental implant and augmentation procedures. Success depends on thorough implant planning, minimally invasive surgery, a detailed medical history review, appropriate pre- and post-surgical medication, and the use of less toxic immunosuppressive drugs. Regular checkups, professional oral hygiene, and patient motivation are crucial for achieving optimal outcomes. Further research is needed to deepen our understanding of implant treatment in immunocompromised patients.
